# Applying Active Learning to the Screening of Molecular Oxygen Evolution Catalysts

**DOI:** 10.3390/molecules26216362

**Published:** 2021-10-21

**Authors:** Michael John Craig, Max García-Melchor

**Affiliations:** CRANN and AMBER Research Centres, School of Chemistry, Trinity College Dublin, College Green, Dublin 2, Ireland

**Keywords:** water splitting, oxygen evolution reaction, machine learning, catalyst design, scaling relations

## Abstract

The oxygen evolution reaction (OER) can enable green hydrogen production; however, the state-of-the-art catalysts for this reaction are composed of prohibitively expensive materials. In addition, cheap catalysts have associated overpotentials that render the reaction inefficient. This impels the search to discover novel catalysts for this reaction computationally. In this communication, we present machine learning algorithms to enhance the hypothetical screening of molecular OER catalysts. By predicting calculated binding energies using Gaussian process regression (GPR) models and applying active learning schemes, we provide evidence that our algorithm can improve computational efficiency by guiding simulations towards candidates with promising OER descriptor values. Furthermore, we derive an acquisition function that, when maximized, can identify catalysts that can exhibit theoretical overpotentials that circumvent the constraints imposed by linear scaling relations by attempting to enforce a specific mechanism. Finally, we provide a brief perspective on the appropriate sets of molecules to consider when screening complexes that could be stable and active for this reaction.

## 1. Introduction

With improved computing power and increased accessibility to said power, large-scale simulations in computational catalysis have become more feasible. This has paved the way for deriving statistical knowledge from quantum chemical simulations, which has opened a new era of data-driven catalyst discovery. Recent examples include the elucidation of a CO_2_ electroreduction catalyst [1] and a spinel oxygen evolution reaction (OER) catalyst [2]. To the best of our knowledge, these catalyst discoveries have been limited to heterogeneous systems, and in each case, machine learning (ML) was applied to a single continuous variable. In this communication, we outline how ML can be applied to multiple relevant OER intermediates in homogeneous systems while incorporating information about the mechanism for M-O bond activation that we have gathered from a previous work [3].

Studying OER mechanisms is more straightforward in molecular catalysts than in heterogeneous systems since the nature of the active site is less ambiguous. An exciting prospect for these molecular catalysts is that, due to their inherent three-dimensional nature, they offer greater flexibility with which to attempt to circumvent scaling relations that limit their activity [4]. This circumvention could occur through geometric effects from a second or third coordination sphere [5,6], a concrete demonstration of which seems to have appeared recently from Llobet and co-workers [7]. Significant challenges remain, however, and a mononuclear first-row transition metal catalyst with comparable activity to Ru or Ir complexes is yet to be found. This is due, in part, to issues relating to stability, although it is not clear a priori why first-row mononuclear catalysts cannot exhibit turnover frequencies matching those of Ru or Ir. As an avenue to investigate this, we have recently proposed that earth-abundant Cr, Mn and Fe-based catalysts could exhibit low overpotentials for this reaction, assuming they undergo an extra oxidation mechanism [8]. To efficiently search such possibilities, ML algorithms can enable an efficient exploration of possible candidate molecules through active learning (AL).

Herein, we set out to showcase a data-driven approach to homogeneous catalyst discovery using an ML-based surrogate function to suggest promising complexes based on intermediate binding energies along the water nucleophilic attack (WNA) mechanism. We restrict our analysis to intermediates that precede the O-O bond formation, since that step is best studied using explicit solvent. Such ML-based approaches must be flexible enough to discover catalysts that evolve oxygen through distinct mechanisms, which may require distinct objective functions while using the same surrogate function. With this premise in mind, we inspect how to use surrogate functions that predict OER descriptors to optimize catalysts for this reaction. Through an expansion of the data acquired in our recent manuscript [3], we set out to design ML algorithms with the intention of making a surrogate model to guide future calculations with AL. Due to the modest size of our dataset, we employed Gaussian processes (GPs) to our problem. These models define prior probability distributions over functions that predict an important value which is later used to construct posteriors by sampling examples. Some applications of GPs include Gaussian process regression (GPR), which has seen application in optimizing the nudged-elastic band method [9], predicting solubility parameters [10], nanoparticle alloy composition [11], redox-flow battery couples [12] and Pourbaix diagrams [13]. However, to the best of our knowledge, these methods have not been applied to homogeneous OER catalysts [14]. For further details on GPs and our implementation, we refer the reader to Refs. [15,16] and the Computational Methods section, respectively.

## 2. Results and Discussion

### 2.1. Machine Learning Models

To represent the modelled OER catalysts, we used reduced autocorrelation (RAC) functions taken from the molSimplify-generated HO* intermediate [17]. First described by Kulik et al. [18], this vectorial representation of molecules is graph based and describes how the individual atoms of a molecule relate to atoms in the *n*th coordination sphere. This method has shown success in predicting spin-splitting and metal-oxo formation energies [18,19]. Vector features for each catalyst are then made from multiplication and subtraction of continuous-valued atomic properties, P, namely electronegativity, covalent radius, polarizability and nuclear charge of a given set of atoms at a given bond-wise distance or depth, d, as shown in Equation (1).

(1) $$P_{md}=\sum_{i}\sum_{j}P_{i}P_{j}\delta \left(d_{ij},\;d\right);\;\;P_{sd}=\sum_{i}\sum_{j}(P_{i}-P_{j})\delta \left(d_{ij},\;d\right)$$

where δ is the Kronecker delta function and $d_{ij}$ is the bond-wise distance between atoms i and j. These indices are chosen such that they are either metal-centred, so that i is fixed as the metal atom index, or ligand-centred, so that i runs over atoms in the first coordination sphere of the metal. We take the unoptimized geometries since we are mimicking a situation wherein we do not have the DFT data at our disposal. However, the values of the features defined in Equation (1) may be sensitive to flexible ligand frameworks.

### 2.2. Active Learning Applied to the OER Descriptor

**Figure 1:**
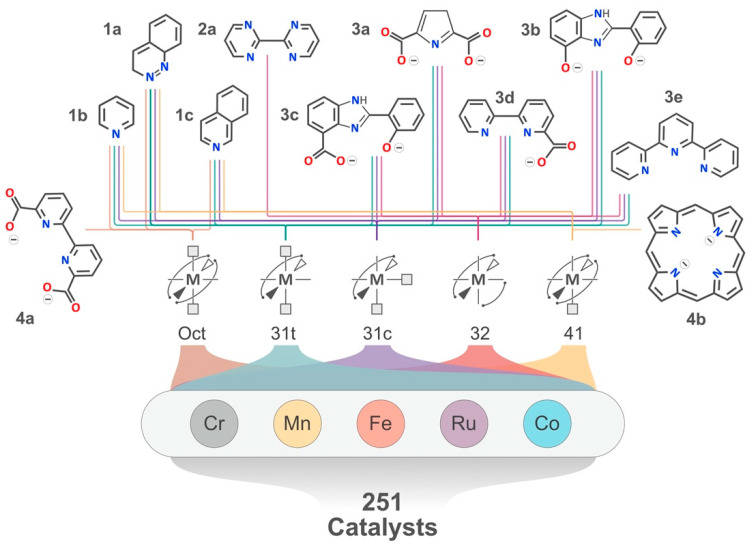
Illustration of the set of catalysts considered to create the datasets required to perform active learning, adapted from Ref. [3] under the terms of the Creative Commons CC-BY license. Each ligand is labelled first by their denticity (i.e. 1, 2, 3 or 4) along with a letter suffix (i.e. a, b, c, d, or e) to distinguish ligands with the same denticity. Monodentate ligands in each of the geometries are represented by grey squares, while the free lines protruding from the metal represent the active site. Where there are two monodentate ligands, they can either be in *cis* or *trans* to each other, leading to the labels 31c or 31t, respectively. The 41 geometry contains the porphyrin ligand 4a along with one of the three monodentate ligands.

Firstly, we aim to use ML to predict the OER descriptor, $\Delta G_{O{\left(IV\right)}^{*}}-\Delta G_{HO{\left(III\right)}^{*}}$, since this descriptor is known to be one of the most descriptive binding energy values and has recently been used as a correlate of the ‘kink’ potential at which a Tafel slope transition occurs for transition metal oxides [20]. For this, we have increased our dataset of OER descriptors from our previous work [3], while using the same subset of catalysts to generate 251 catalyst OER descriptor pairs, as shown in Figure 1. To enable ML for the OER descriptor, we created feature vectors from Equation (1) using unoptimized cartesian coordinates of the catalysts, so that the speed of predictions on new complexes using ML increases by orders of magnitude over density functional theory (DFT) methods.

**Figure 2:**
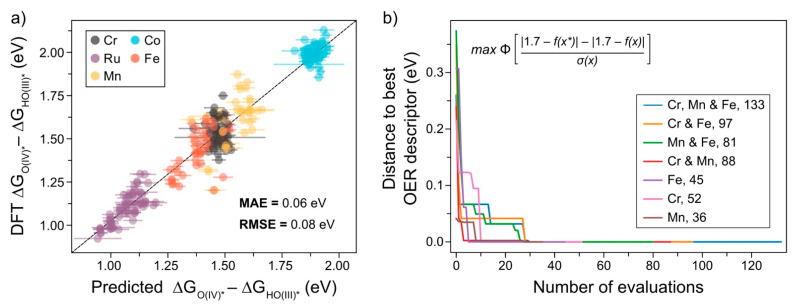
Machine learning applied to OER catalysts: (**a**) results of leave-one-out cross validation using the GPR model for each $\Delta G_{O{\left(IV\right)}^{*}}-\Delta G_{HO{\left(III\right)}^{*}}$ descriptor value. The dashed line represents the equation y=x, while the uncertainties in the predictions are represented as x-axis error bars. (**b**) Bayesian optimization starting from different sets of unseen data, showing the number of evaluations needed to converge to the desired $\Delta G_{O{\left(IV\right)}^{*}}-\Delta G_{HO{\left(III\right)}^{*}}$ value as dictated by the probability of improvement acquisition function, depicted in the top left. When maximized, this function iteratively determines the most promising catalyst to evaluate. This equation and the terms are explained in the main text.

To reduce overfitting, and since our dataset is modest in size, we have applied leave-one-out cross validation (LOOCV) to evaluate the performance of the GP model. This means that the OER descriptors are predicted using 251 different training and test sets, so that each catalyst is evaluated as its own test set. To determine the form of the RACs to represent catalysts, we have used a grid search over the space of metal-centred depths ranging from 2 to 4, and ligand-centred depths of either 0 or 1 (see Equation (1)). Based on the result of each combination (Figure S1), we have taken the combination that produces the lowest error while leading to the minimum number of features, which corresponds to a metal-centred depth of 3 and a ligand-centred depth of 0. Notably, our model, shown in Figure 2a, produces a LOOCV mean absolute error (MAE) of only 0.06 eV and a root mean square error (RMSE) of 0.08 eV, respectively, which is within the error of DFT calculations and within previous standard deviations of cross-functional binding energies for heterogeneous OER and ORR catalysts [21]. We note, however, that applying a coarse baseline model that simply predicts the OER descriptor as the mean value for a given set of metals provides a similar MAE and RMSE, i.e., 0.08 and 0.10 eV, respectively. The data and analysis of the influence of individual features on the performance of this procedure are presented in Table S1 and in the “Feature importance” section of the Supplementary Information. This implies that the covalent radii and the features generated at a bond distance of 1 are the most important features for this approach. Yielding more descriptive design rules, however, will necessitate the generation of a larger dataset, which is not the focus of this communication. In addition to this, we have tested support vector regression, random forest regression and kernel ridge regression with hyperparameter optimization in an attempt to reduce the errors further. In Table S2, we present the best result for a given algorithm after hyperparameter tuning over the primary parameters for each algorithm, with support vector regression and random forest regression exhibiting a similar error to the GPR to the first significant figure. Yet, we have opted to use GPR over these other models since they provide mathematically-derived uncertainty estimates along with predictions, which can be exploited by acquisition functions to perform Bayesian optimization on the OER descriptor as part of AL, as we describe in the following. One foreseen drawback of GPR methods, however, is their poor $O\left(n^{3}\right)$ scaling, which will be a limitation for a higher volume of data. If this becomes prohibitive, we may utilize a scheme to handle uncertainty quantification using deep learning methods, as has been recently described by Kulik and co-workers [22].

Herein, we have applied Bayesian optimization through the surrogate GPR model function, f, which can be evaluated with ease compared to DFT, and it approximates the OER descriptor value well. Then, knowing the current best value, $f^{*}$, one can maximize an acquisition function, μ, which approximates the probability that the evaluated catalyst has a more favourable OER descriptor than the current best $f^{*}$. In our case, we define the best $\Delta G_{O{\left(IV\right)}^{*}}-\Delta G_{HO{\left(III\right)}^{*}}$ to be 1.7 eV, based on the scaling relation shown in Figure S2, which identifies this value to be the optimal range when complexes are constrained by the established scaling relation, although the best value can be tailored to the type of mechanism one wishes to optimize, as we outline later.

To test the ability of this model to perform Bayesian optimization, we have applied the probability of improvement (PI) acquisition function:

(2) $$\mu \left(\vec{x}\right)=\Phi \left(\frac{\left|1.7-f\left({\vec{x}}^{*}\right)\right|-\left|1.7-f\left(\vec{x}\right)\right|}{\sigma \left(\vec{x}\right)}\;\right)$$

where, $\vec{x}$ represents the feature vector of a given catalyst, σ is the uncertainty from the GPR, Φ is the cumulative distribution function of a normal distribution, ${\vec{x}}^{*}$ represents the feature vector of the catalyst that has a value for the OER descriptor closest to the optimum (i.e., 1.7 eV) and $f\left(\vec{x}\right)$ is the GPR prediction for $\vec{x}$.

The results of performing Bayesian optimization on sets of unseen catalyst data composed of different combinations of the desirable Cr, Mn and Fe metals are presented in Figure 2b. To simulate what would be conducted in future screening studies, we have fitted our ML model without exposing it to each combination of abundant metals, and re-fitting individual catalyst datapoints when Equation (2) suggested that we evaluate that catalyst. In each case, the optimization strategy converges to the best of the best possible candidates in less than 30% of the total number of evaluations. We compare this to an approach using random forest regression, where the uncertainty is defined as the standard deviation of each estimator. However, in many cases, using this approach does not lead to convergence to the best value within 50%, as seen in Figures S3 and S4, using the probability of improvement and expected improvement acquisition functions, respectively. The GPR approach, therefore, represents a promising route to optimize catalysts for distinct catalytic properties, since one can choose the desired OER descriptor arbitrarily. In addition, we have tested the performance of this acquisition function to the same procedure using the expected improvement (EI) acquisition function by comparing the cumulative regret of the AL procedure (see Supplementary Materials). This is a common measure of AL performance, which measures the difference between the best possible value available to the AL scheme and the value suggested by the scheme over the course of the AL procedure. The results of this for the two acquisition functions are shown in Figure S5. We note there are only minor differences in this metric between the two acquisition functions, but the EI acquisition function does not converge to the optimum catalyst as fast as the PI function for the Cr, Fe combination (see Figure S6).

In the rest of the communication, we use the results in Figure 2 as a proof of concept of this Bayesian optimization strategy and assume similar results will be achieved for other descriptor values, provided enough data are available. Assuming performant GPR models for these steps, which precede O-O bond formation, we envision two approaches for optimizing molecular OER catalysts via AL. The first one involves honing in on the ideal OER descriptor, as we have described above, while the other focuses on optimizing oxygen evolution via an extra oxidation mechanism [8], which we outline in the following.

### 2.3. AL for an Extra Oxidation Mechanism

Another AL approach that we could apply to our catalysts aims to find catalysts with overpotentials that are not dictated by scaling relations, but instead evolve oxygen via an extra oxidation mechanism [8]. For this, we attempt to strike a balance between having a low overpotential and having a proton transfer barrier, which makes fast OER feasible. We know from the scaling shown in Figure S2 that $\Delta G_{HOO{\left(III\right)}^{*}}-\Delta G_{HO{\left(III\right)}^{*}}\approx 3.4$ eV, and that if we are to evolve oxygen via the extra oxidation mechanism, there will be a proton transfer step from M(V)-O to M(III)-OOH. This chemical step, defined in terms of binding energies as $\Delta G_{HOO{\left(III\right)}^{*}}-\Delta G_{O{\left(V\right)}^{*}},$ effectively determines what values of $\Delta G_{O{\left(IV\right)}^{*}}-\Delta G_{HO{\left(III\right)}^{*}}\;$and $\Delta G_{O{\left(V\right)}^{*}}-\Delta G_{O{\left(IV\right)}^{*}}$ are desirable, (i.e., $(\Delta G_{O{\left(IV\right)}^{*}}-\Delta G_{HO{\left(III\right)}^{*}})/2$), leading to the possibility of overpotentials lower than those imposed by the scaling. While lower overpotentials are of course desirable, we also need to concern ourselves with the proton transfer step. Tangible evidence for this is provided by our recent computational results using the same DFT methodology for an amidate-ligated Fe OER catalyst [23], for which we predicted an extremely low overpotential of 0.08 V, and a proton transfer barrier of 1 eV [3]. This corroborates experiments, as the catalyst exhibits extremely low overpotential of 0.02 V, while also showing a very low turnover number [23]. We therefore set a value that we propose to be achievable for the proton transfer, i.e., $\Delta G_{HOO{\left(III\right)}^{*}}-\Delta G_{O{\left(V\right)}^{*}}=$ 0.5 eV. This value is chosen so that we can find catalysts with overpotentials of at least 220 mV, while exhibiting a realizable proton transfer step; however, the choice of $\Delta G_{HOO{\left(III\right)}^{*}}-\Delta G_{O{\left(V\right)}^{*}}$ value is to some extent arbitrary. Concretely, then, we want the following three conditions to hold:

(3) $$\Delta G_{O{\left(IV\right)}^{*}}-\Delta G_{HO{\left(III\right)}^{*}}=1.45\;\mathrm{eV}$$

(4) $$\Delta G_{O{\left(V\right)}^{*}}-\Delta G_{O{\left(IV\right)}^{*}}=1.45\;\mathrm{eV}$$

(5) $$\Delta G_{O{\left(IV\right)}^{*}}<\Delta G_{HO{\left(IV\right)}^{*}}$$

where the conditions expressed in Equations (3) and (4) ensure that the energies of the elementary steps preceding M(V)-O are equally distributed between M(V)-O and M(III)-OH, as (3.4−0.5)/2=1.45 eV, and the condition in Equation (5) ensures that the extra oxidation mechanism occurs, as opposed to the mechanism involving only the traditional OER descriptor.

If the condition in Equation (5) is met, then:

(6) $$\Delta G_{O{\left(IV\right)}^{*}}-\Delta G_{HO{\left(III\right)}^{*}}<\Delta G_{HO{\left(IV\right)}^{*}}-\Delta G_{HO{\left(III\right)}^{*}}$$

Given that we have established scaling relations for Mn and Fe, which are oxidation state independent [3] for a given metal, we can make the following approximations:

(7) $$\Delta G_{O{\left(V\right)}^{*}}=m_{M}\Delta G_{HO{\left(IV\right)}^{*}}+c_{M}$$

(8) $$\Delta G_{O{\left(IV\right)}^{*}}=m_{M}\Delta G_{HO{\left(III\right)}^{*}}+c_{M}$$

where $m_{M}$ and $c_{M}$ denote the slope and intercept, respectively, for a given metal determined by scaling relations. The assumption of these values is a limitation of this approach and may require an on-the-fly update of the scaling relations through calculation of the ‘vacancy’ intermediate for a subset of catalysts. In any case, assuming fixed $m_{M}$ and $c_{M}$, we arrive at the expression:

(9) $$\Delta G_{O{\left(V\right)}^{*}}-\Delta G_{O{\left(IV\right)}^{*}}\approx m_{M}\left(\Delta G_{HO{\left(IV\right)}^{*}}-\Delta G_{HO{\left(III\right)}^{*}}\right)$$

**Figure 3:**
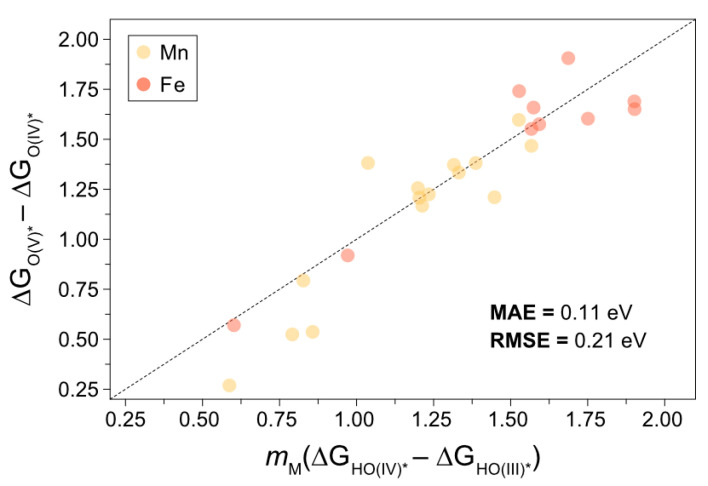
Parity plot showing that Equation (9) holds for Fe and Mn catalysts considered in this work using the scaling relations presented in Figure S7 with $m_{M}$ values of 1.18 and 1.36 for Fe and Mn, respectively. The dashed line denotes the equation y=x.

The demonstration of this relationship described in Equation (9) is presented in Figure 3, and the scaling relations used to arrive to the values for $m_{M}$ are shown in Figure S7.

With the relationship described in Equation (9), we can use our ML models to begin to formulate an AL strategy aiming to meet the conditions imposed in Equations (3)–(5). That is, given the predicted $\Delta G_{O{\left(IV\right)}^{*}}-\Delta G_{HO{\left(III\right)}^{*}}$ and $\Delta G_{O{\left(V\right)}^{*}}-\Delta G_{O{\left(IV\right)}^{*}}$ values, we can prioritize those catalysts that are forecast to satisfy such conditions. To achieve this, we propose the following acquisition function, which we will aim to maximize:

(10) $$\Phi \left(\frac{\left|1.45-f_{1}\left({\vec{x}}^{*}\right)\right|-\left|1.45-f_{1}\left(\vec{x}\right)\right|}{\sigma_{1}\left(\vec{x}\right)}\right)\Phi \left(\frac{\left|1.45-f_{2}\left({\vec{x}}^{*}\right)\right|-\left|1.45-f_{2}\left(\vec{x}\right)\right|}{\sigma_{2}\left(\vec{x}\right)}\right)\left(\frac{1}{m_{M}}f_{2}\left(\vec{x}\right)-f_{1}\left(\vec{x}\right)\right)$$

**Figure 4:**
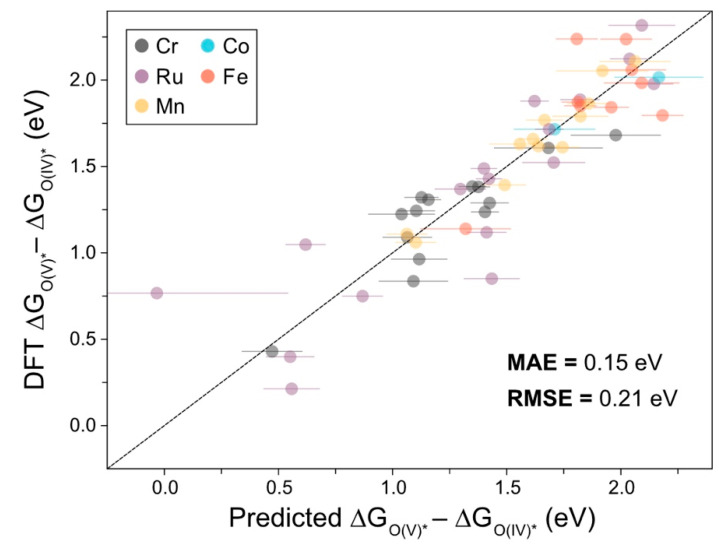
Parity plot for the LOOCV predictions of $\Delta G_{O{\left(V\right)}^{*}}-\Delta G_{O{\left(IV\right)}^{*}}$ for a subset of catalysts which were considered in Figure 2a, with MAE and RMSE values shown.

Note that, in Equation (10) we used the same definitions as in Equation (2), and we further defined $f_{1}$ and$\;\sigma_{1}\;$as the outputs of the GPR model predicting $\Delta G_{O{\left(IV\right)}^{*}}-\Delta G_{HO{\left(III\right)}^{*}}$ and $f_{2}$ and $\sigma_{2}\;$as the outputs of the model predicting $\Delta G_{O{\left(V\right)}^{*}}-\Delta G_{O{\left(IV\right)}^{*}}$. The value of 1.45 eV in the cumulative distribution function of a normal distribution is chosen to satisfy Equations (3) and (4), while the final value in the product is used to satisfy Equation (6). With these premises, we have developed the preliminary model to predict $\Delta G_{O{\left(V\right)}^{*}}-\Delta G_{O{\left(IV\right)}^{*}}$, shown in Figure 4. Note that we do not carry out Bayesian optimization for this procedure since we do not have enough examples of Mn or Fe catalysts with $\Delta G_{O{\left(V\right)}^{*}}-\Delta G_{O{\left(IV\right)}^{*}}$.

The calculated MAE for the $\Delta G_{O{\left(V\right)}^{*}}-\Delta G_{O{\left(IV\right)}^{*}}$ predictions in Figure 4 is larger than the model represented in Figure 2a (0.15 eV vs. 0.06 eV). However, the predictions in Figure 4 are much more performant than the baseline approximation, which forecasts the descriptor value to be the average value for a given metal (i.e., the baseline model gives a MAE = 0.40 eV). This demonstrates that these models can generalize and predict these energies with reasonable accuracy. The criteria outlined in Equations (3)–(5) could, for example, be generalized to higher oxidation states, since it has been shown that Fe(VI) states could be important in catalyzing water splitting for NiFe oxyhydroxides [24]. Maximizing Equation (10) amounts to optimizing the redox potentials approach M(V)-O from M(III)-OH, but to generalize to M(VI)-O, we would need to start from M(IV)-OH; otherwise, there are more intermediates involved to consider, and creating an acquisition function (or functions) to handle this is outside the scope of this communication.

Finally, we note that this AL approach does not tackle the kinetics involved in the O-O bond formation itself, often thought to correlate with the binding energy of the HOO* intermediate. While we focus on the steps before this, optimizing the HOO* binding energy, given appropriate WNA intermediate energies preceding this step, is an exciting prospect. This could be achieved, for example, by tuning the metal ligands or by designing complexes so that the M(V)-O intermediate is flexible enough to selectively bias O-O bond formation, although this is outside the scope of the present work.

### 2.4. Dataset Bias

**Figure 5:**
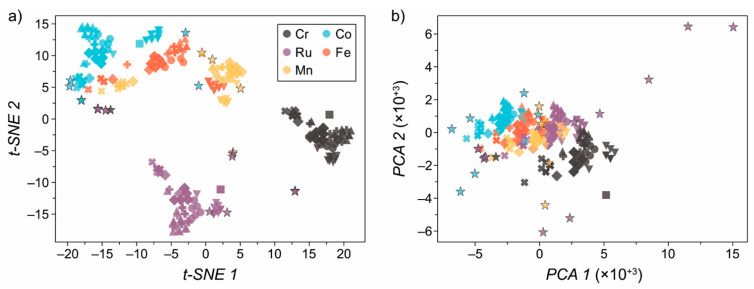
Dimensionality reduction via (**a**) t-SNE and (**b**) PCA analyses applied to the set of complexes under study. Points denoted by a star and black outline are taken from the *tmqm* dataset [29], which is a representative slice of the Cambridge Structural Database. Note that these data points are not included in Figure 2a.

We now inspect how our current dataset leads our model to be overfit to the set of complexes we previously studied [3]. The ligands in our original high-throughput study were chosen since they were found in active Ru-based complexes, meaning the dataset was implicitly biased towards this type of catalyst. This presents a challenge as well as an opportunity for creating a balanced dataset. To demonstrate that there is a far larger space of transition metal chemistry to explore, in Figure 5a,b, we present the t-distributed stochastic neighbour embedding (t-SNE) [25,26] and principle component analysis (PCA) [27] dimensionality reduction techniques, respectively, applied to the RACs used to train our GPR model. For further details on t-SNE and PCA techniques, see Refs. [25,28], respectively. In short, using these dimensionality reduction techniques, we can reduce the size of our feature vector space to a two-dimensional space that we can visualize. We then compare the RACs from our study to a comparable set of RACs that we could expect to see in future studies. To form this representative set of complexes, we have used the *tmqm* dataset [29], which includes a subset of mononuclear complexes taken from the well-known Cambridge Structural Database (CSD) [30]. Within this dataset, we searched for either Cr, Mn, Fe, Co or Ru octahedral complexes which contain an HO* adsorbate. The choice of the HO* adsorbate was made to allow a fair comparison to the original RACs.

From Figure 5, we can indeed observe that the distance between the space of complexes, which represents only a small sliver of the entire chemical space, shows that there are more catalysts to explore. In particular, we note that, for each metal, there are points from the *tmqm* dataset that lie far away from the set of catalysts we have studied; hence, we can assume that our GPR will have low confidence. This impels the generation of balanced datasets while performing AL by assessing the feature space prior to evaluating a given catalyst so that there are no similar complexes. This could be mitigated by the acquisition functions Equations (2) and (10), since the uncertainty measures will correlate to the prior exposure of the model to similar structures, i.e., the more uncertain the model is, the larger the acquisition function value is, by design.

### 2.5. Outlook

In the final part of this communication, we propose that the space of ligands that could make up effective OER catalysts must fulfil certain criteria, which will help to constrain our search. Firstly, we posit that metal ligands must be multidentate to handle the lability of first-row transition metal complexes; multiple monodentate ligands are likely to become hydrolysed, so the appropriate catalyst to model in this case would be some MO_x_H_y_-type catalyst. While utilizing predominantly monodentate or bidentate ligands allows for greater combinatorial flexibility and much larger datasets, their inclusion is not realistic for the labile first-row transition metal with which we want to use to design active catalysts. Secondly, any organic ligand framework proposed for water oxidation must also be oxidatively stable. An inspiring and insightful overview of these considerations was recently outlined in a perspective by Nocera and Thorarinsdottir [31]. Here, we highlight two of the useful instructions that the authors summarized from seminal works by Collins [32], outlining an instructive ruleset for making oxidatively stable organic ligands: “(1) elimination of β-H atoms, especially if the α atom can support an increase in bond order with β-H elimination; (2) elimination of heteroatoms that can stabilize the cationic character that remains on atoms from which oxidative bond cleavage has occurred”. We highlight these considerations specifically since ligands can be filtered computationally on this basis by creating code that can distinguish types of H atoms and iteratively apply point (2). Concrete demonstrations of the first rule for molecular OER were provided by Fillol and co-workers reporting a five-fold improvement in turnover number after deuterating β-H atoms [33]. Furthermore, the same authors also showed that deuteration of methyl groups could lead up to a ca. 10-fold improvement in turnover frequency. This was proposed to be due to C-H hydroxylation whereby the H atom was transferred to the Fe(V) = O site. In this context, our recent computational insights suggested the importance of having at least a 3.0 A distance between the WNA active site and the most proximal methyl group [34]. In any case, tight collaboration between computational and inorganic chemists is required to realize the potential of any endeavour to create a useful and applicable search space of OER catalysts.

## 3. Materials and Methods

DFT calculations and the calculation of binding energies reported in this work were carried as described in Ref. [3] using the meta-GGA functional TPSSh [25], as implemented in Gaussian09 [35]. To describe the Ru, Mn, Fe, Cr and Co metals, the Lanl2dz effective core potential was used, along with *f*-polarization functions, with exponents 1.235, 2.195, 2.462, 1.941 and 2.78, respectively [36]. The more electronegative O and N atoms were described using the 6–31+G(d) basis set, and the 6–31 g(d,p) basis set was used for C and H atoms. Molecular structures were optimized in water (ε = 78.3553) with the implicit SMD solvation model [37]. Gibbs energies were calculated at the temperature of 298.15 K and pressure of 1 atm, except for the isolated H_2_O molecule that was computed at the temperature and pressure at which both the liquid and gas phases were in equilibrium, i.e., 300 K and 0.035 atm. Relative Gibbs energies are referenced to H_2_O and H_2_ in solution to avoid introducing the error associated with the modeling of O_2_ with DFT methods, and the global reaction Gibbs energy was fixed to the experimental value of 4.92 eV. To ensure sound geometries, we inspected any intermediate where atoms coordinated to the metal change or where a bond distance changed by 20% or more. The ML analysis was repeated using the TPSSh-optimized geometries to ensure that the results and conclusions remained salient using differing geometries. These results can be found in Figures S8–S10 in the “Model sensitivity to input geometries” section in the Supplementary Materials. Grimme D3 dispersion corrections [38] were added via single-point calculations at the optimized geometries.

To perform Gaussian process regression, we used scikit-learn [39]. The kernel was described using a scaled squared exponential kernel, with a noise of 0.01 added to the diagonal of the kernel, which was increased so that the model could converge. The bounds for this kernel were increased with respect to the default value from scikit-learn to optimize the RMSE. Each RAC feature vector was scaled such that it had a mean of 0 and variance of 1. We also use scikit-learn for random forest regression, kernel ridge regression and support vector regression. The software optuna [40] was used to optimize hyperparameters, with the search space defined in the section titled “Hyperparameter search” in the Supplementary Materials.

## 4. Conclusions

In this communication, we have outlined a preliminary AL scheme to be applied in the screening of homogeneous OER catalysts. The proposed scheme uses a surrogate GPR model to predict binding energies, which thereby guide future calculations by optimizing for either the ideal OER descriptor, or for optimized oxygen evolution via the extra oxidation mechanism, using previously derived scaling relations to guide AL strategies. This model can be applied to force field-optimized geometries and is therefore orders of magnitude faster than present-day DFT at predicting relevant OER binding energies. It is also noteworthy that individual metals may require individual screening strategies that account for the constraints imposed by scaling relations, instead of assuming universal descriptors across metal centers. The challenges and outlook for generating realizable and useful datasets with which to apply these AL strategies have also been outlined and discussed, which are expected to be useful to future screening studies in the homogeneous OER domain. These studies should utilize ML models to guide computational simulations as described in this communication. This is in part because it will allow faster discovery, but also because it will avoid simulations that are needless and time consuming. It must be remembered that computing time has an associated carbon footprint [41], which should be minimized where possible. AL schemes such as the ones we have put forward aim at reducing this burden and can be easily applied to heterogeneous OER studies.

## Data Availability

All calculated structures and energies can be found at the ioChem-BD repository https://iochem-bd.bsc.es/browse/handle/100/198436 (accessed 19 October 2021). The binding energies of all the OER intermediates calculated with TPSSh can be found in Data S1 as DataS1.xlsx. Software required to carry out the analyses presented can be found at https://github.com/michaelcraiger/oer_active_learning (accessed 19 October 2021). This includes code to check geometries, create the RACs, perform grid searches over the RAC depths, analyze other machine learning models using optuna, evaluate the OER descriptors using Gaussian process regressors, and perform Bayesian optimization over the OER descriptors.

## Supplementary Materials

The following are available online: The results of applying GPR to differing combinations of RAC metal and ligand-centered depths is outlined (Figure S1); the scaling relations used to derive the acquisition functions (Figures S2 and S7); feature importance (Table S1); hyperparameter search and details for the performance of standard ML algorithms (Section Hyperparameters Search and Table S2, respectively); random forest regression applied to Bayesian optimization using PI and EI acquisition functions (Figures S3 and S4, respectively); comparisons of PI and EI acquisition functions by comparing cumulative regret (Figure S5); the performance of applying EI acquisition function mirroring the results shown in Figure 2b of the main text (Figure S6); metal-dependent scaling relations (Figure S7); the performance of the models using TPSSh-optimized geometries (Figures S8–S10) and details of the energies and cartesian coordinates are provided in the Supplementary Materials.

## Sample Availability

Samples of the compounds are available from the authors.
