# Supplementary material for: Applying Active Learning to the Screening of Molecular Oxygen Evolution Catalysts
Source: Molecules. 2021 Oct 21;26(21):6362. doi: 10.3390/molecules26216362 (PMC8588390; doi:10.3390/molecules26216362)
Supplement: Supplementary file 1 [file molecules-26-06362-s001.zip › molecules-1386849-supplementary.pdf]

Supplementary Information for:

Applying Active Learning to the Screening of Molecular Oxygen Evolution Catalysts

Michael John Craig,[a*] Max García-Melchor[a*]

[a] School of Chemistry, CRANN and AMBER Research Centres, Trinity College Dublin, College Green, Dublin, Ireland

*Correspondence should be addressed to: craigmi@tcd.ie ; garciamm@tcd.ie

Contents

RAC depth model comparison S2

Feature importance S3

Scaling relation S4

Hyperparameter search S5

Random forest regression for Bayesian optimization S7

Acquisitions functions S9

Metal-dependent scaling relations S11

Model sensitivity to input geometries S12

Cartesian coordinates and energies S16

RAC depth model comparison

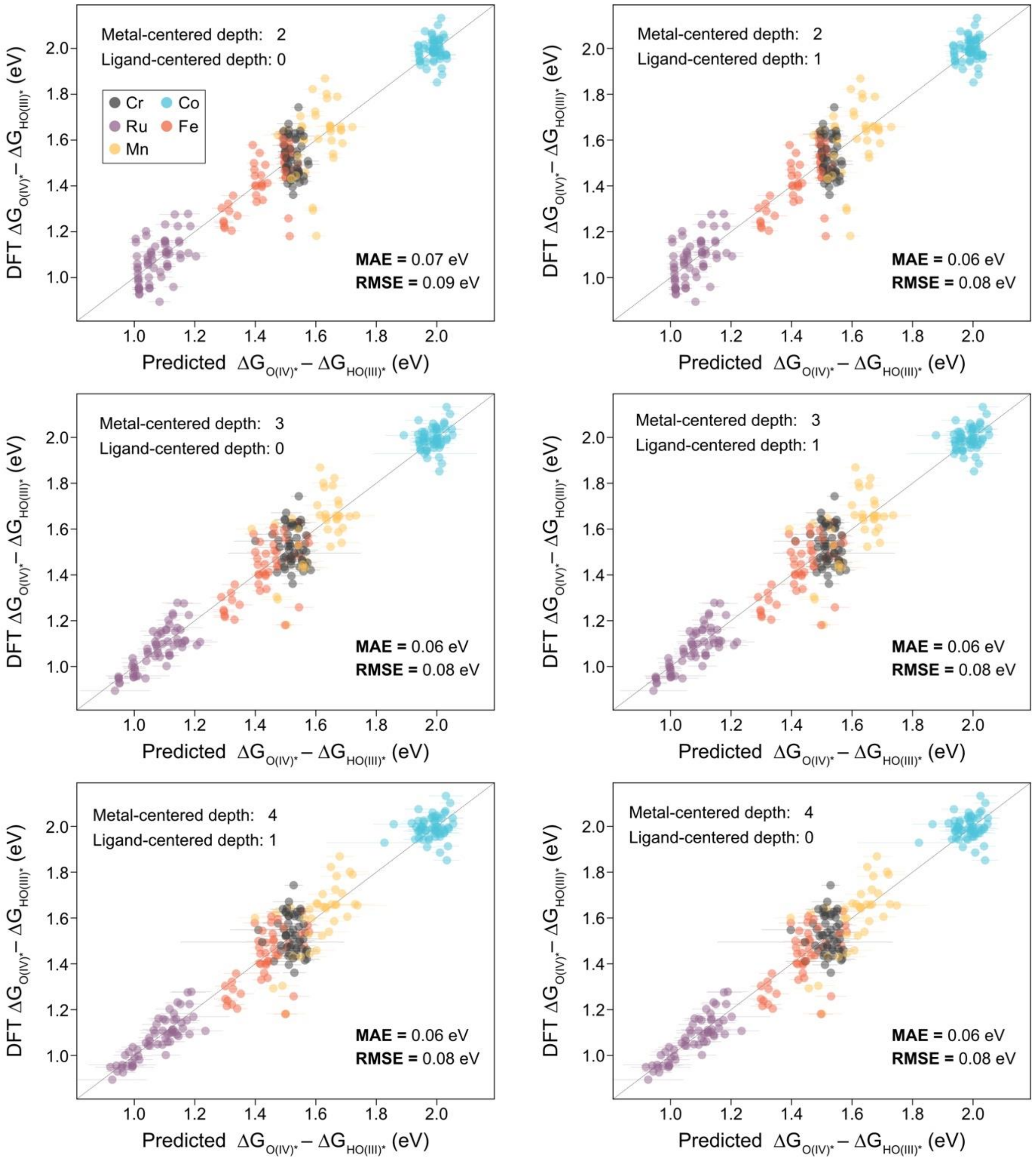

Figure S1. Gaussian process regressor performance for different combination of metal and ligand-centered depths, the figure mirrors that of Figure 2a, with the data in the 3, 0 combination representing the same data as Figure 2a.

Feature importance

We have applied this to the extra oxidation descriptor, $\Delta G_{O(V)^*} - \Delta G_{O(IV)^*}$, since the intra-metal variance is large so that a baseline model predicting the mean value of each metal as a prediction is less performant. Therefore, the feature importance analysis will be more informative.

Table S1. LOOCV error when training the model only on one specific feature set in the RAC.

Feature set	RMSE (eV)	MAE (eV)
Electronegativity	0.98	0.72
Covalent radius	0.29	0.20
Polarizability	0.34	0.26
Nuclear charge	0.57	0.41
All depth 1	0.47	0.34
All depth 2	0.63	0.44
All depth 3	0.88	0.63
All	0.20	0.15

We note that there are issues with this analysis as the degree to which these effects are due to the correlated nature of the variables is not explored. Hence, the transferability of this feature importance results to any general inference about their effects on the binding energy is questionable and may only be applicable to the subset of complexes that are analyzed.

Scaling relation

We use scaling linear scaling relations in the intermediates for the water nucleophilic attack mechanism to define the acquisition functions used for Bayesian optimization. The choice of 1.7 (*i.e.* 3.4/2) eV for the OER descriptor is chosen due to the scaling relation between $\Delta G_{HO(III)^*}$ and $\Delta G_{HOO(III)^*}$, as seen below.

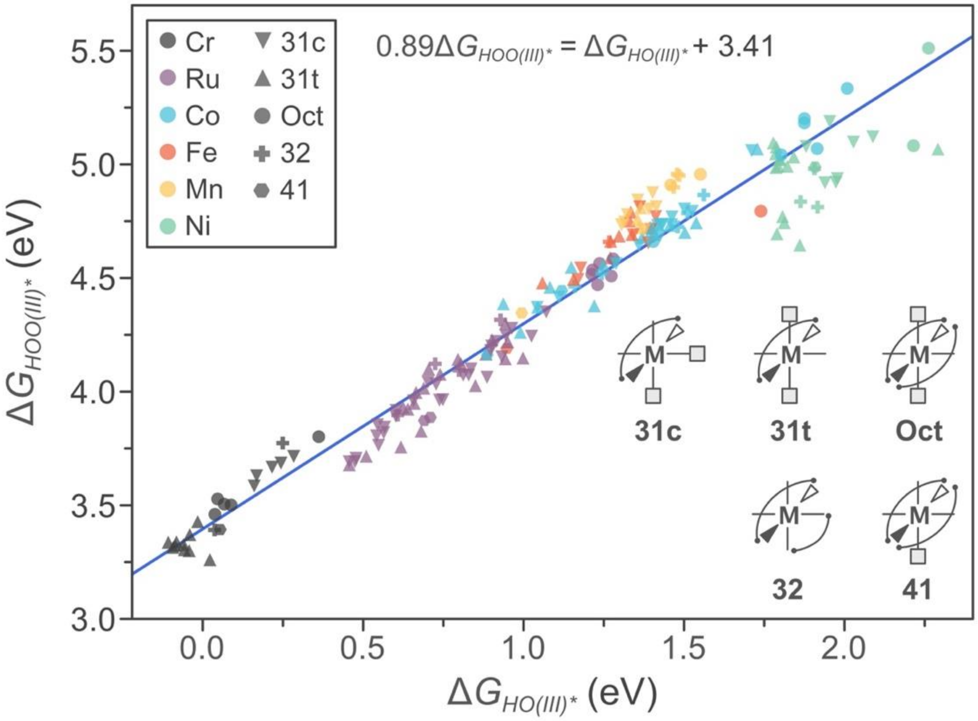

Figure S2. Metal-independent scaling relations for $\Delta G_{HO(III)^*}$ and $\Delta G_{HOO(III)^*}$ as in Ref. 3 from the main text. The intercept of 3.41 eV means the ideal OER descriptor is approximately 1.7 eV.

Hyperparameter search

To explore other potential methods for use in screening studies, the python package optuna was used. To use this package, we simply initialize the set of models we want to analyze as well as the set of parameters for each individual model. Optuna then uses Bayesian optimization to search for the model and set of parameters which perform best. In our case, we defined the RMSE for the leave one out cross-validation as the metric to optimize. The hyperparameters which we allowed to vary were the primary variables described listed in the scikit-learn.org documentation for a given algorithm. Those parameters, and their associated ranges are shown below as a block of code.

```
import optuna
from sklearn.svm import SVR
from sklearn.ensemble import RandomForestRegressor
from sklearn.kernel_ridge import KernelRidge

# define objective function
def objective(trial):
    # define names of algorithms arbitrarily, but optuna treats this as
    # a categorical variable to optimize.
    regressor_name = trial.suggest_categorical("regressor", ["SVR", "RandomForest",
"KRR_RBF", "KRR_Linear"])

    # value ranges for the hyperparameters of each model
    if regressor_name == "SVR":
        epsilon = trial.suggest_float("epsilon", 1e-3, 1, log=True)
        regularization = trial.suggest_float("reg_svr", 1e-3, 10, log=True)
        regressor_obj = SVR(epsilon=epsilon, C=regularization)
    elif regressor_name == "RandomForest":
        rf_n_estimators = trial.suggest_int("rf_n_estimators", 10, 1000)
        rf_max_depth = trial.suggest_int("rf_max_depth", 2, 32, log=True)
        regressor_obj = RandomForestRegressor(
            max_depth=rf_max_depth, n_estimators=rf_n_estimators, random_state=42
        )
    elif regressor_name == "KRR_RBF":
        krr_alpha = trial.suggest_float("krr_alpha", 0.01, 1000)
        regressor_obj = KernelRidge(kernel="rbf", alpha=krr_alpha)

    elif regressor_name == "KRR_Linear":
        krr_l_alpha = trial.suggest_float("krr_l_alpha", 0.01, 1000)
        regressor_obj = KernelRidge(kernel="linear", alpha=krr_l_alpha)    …
    # here we have code to evaluate models
    …
    return accuracy

study = optuna.create_study(direction=”minimize”)
study.optimize(objective, n_trials=1000)
```

We stopped the optimization when no improvements occurred for a sustained period. The study found the lowest error for the support vector regression with regularization parameter of 4.87 and epsilon value of 0.06. A table with the best parameters for each model, along with the associated RMSE, is seen below.

Table S2. Errors and optimized parameters for each of the models considered by optuna.

**Model**	**RMSE (eV)**	**Optimized Parameters**
SVR	0.08	Epsilon = 0.06, regularization parameter = 4.87
RFR	0.08	No. of estimators (trees) = 21, Max tree depth = 6
KRR Linear	1.53	Regularization parameter (alpha) = 39.95
KRR RBF	0.49	Regularization parameter (alpha) = 24.33

Full outputs of the Bayesian optimization from optuna can be found at the end of the following Jupyter notebook:
https://github.com/michaelcraiger/oer_active_learning/blob/main/optuna_for_models.ipynb
found in the GitHub repository accompanying this work.

Random forest regression for Bayesian optimization

A random forest regressor is built by creating a number of ‘trees’ which predict a variable based on generating the value which minimizes the mean squared error in a series of sets of training data created with binary decision splits over the features in the training set.

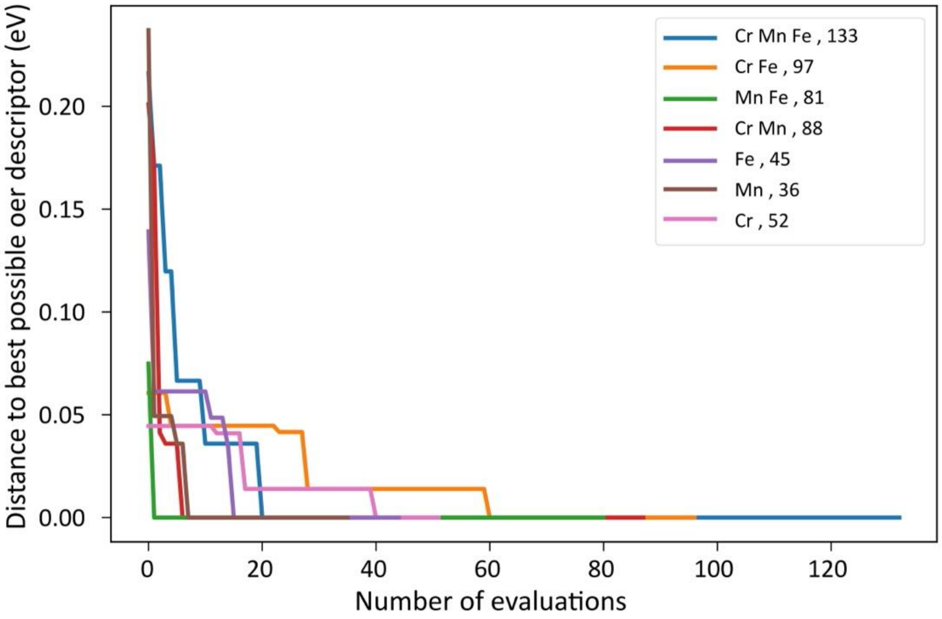

Figure S3. Convergence as in Figure 1b in the main text, using the probability of improvement acquisition function. Here, distinct from the data in Figure 1b, we use random forest regression for predictions and take the standard deviation of the trees of the model as our uncertainty.

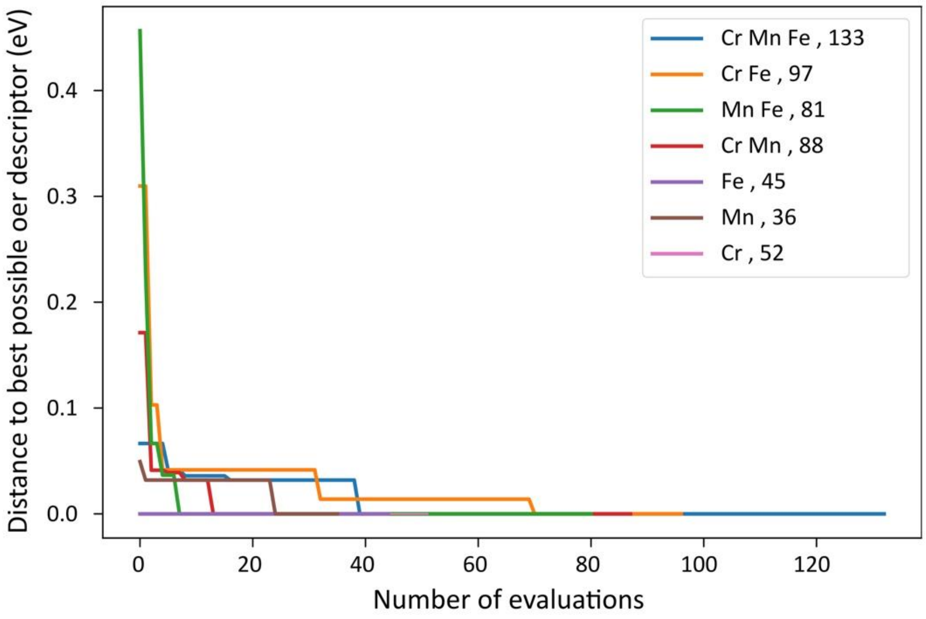

Figure S4. Convergence as in Figure S6, using the expected improvement acquisition function, using random forest regression.

Acquisition functions

To compare the expected improvement and probability of improvement acquisition functions, we have used a commonly used approach known as cumulative regret. The probability of improvement (PI) and expected improvement (EI) acquisitions functions, $\mu_{PI}(x)$, and $\mu_{EI}(x)$, respectively, are shown below:

$$\mu_{PI}(\vec{\boldsymbol{x}}) = \Phi\left(\frac{|1.7-f(\vec{\boldsymbol{x}}^{*})|-|1.7-f(\vec{\boldsymbol{x}})|}{\sigma(\vec{\boldsymbol{x}})}\right); \qquad \mu_{EI}(\vec{\boldsymbol{x}}) = \mu_{PI}(\vec{\boldsymbol{x}}) + \sigma(\vec{\boldsymbol{x}})\varphi(\vec{\boldsymbol{x}})$$

Where $\mu_{PI}(x)$ is defined in the same way as in Eq. 2 in the main text and φ in $\mu_{EI}(\vec{\boldsymbol{x}})$ denotes the probability density function of a normal distribution.

We define cumulative regret as the difference in binding energy between the best possible catalyst to suggest and the one the active learning scheme selected; we track this over the course of the active learning. If we apply this to three different active learning scenarios, corresponding to performing active learning for differing sets of metals, we see that there is only a marginal difference between the two methods.

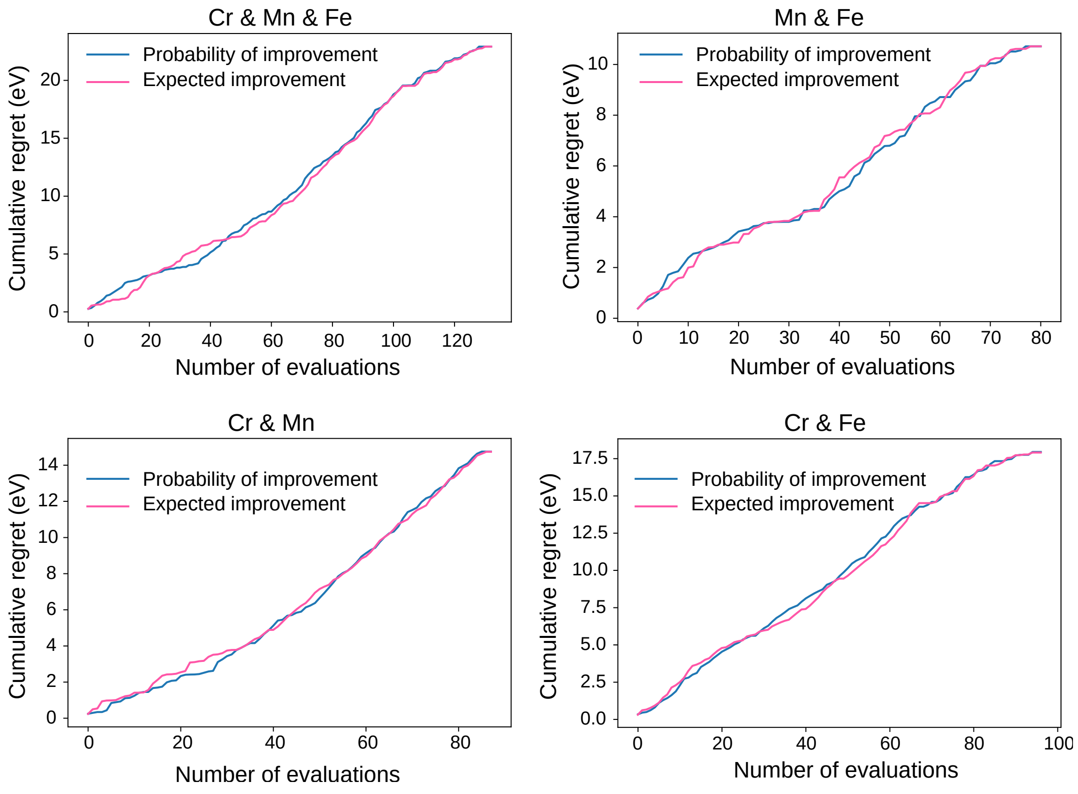

Figure S5. Cumulative regret plots for different sets of catalysts which were held out.

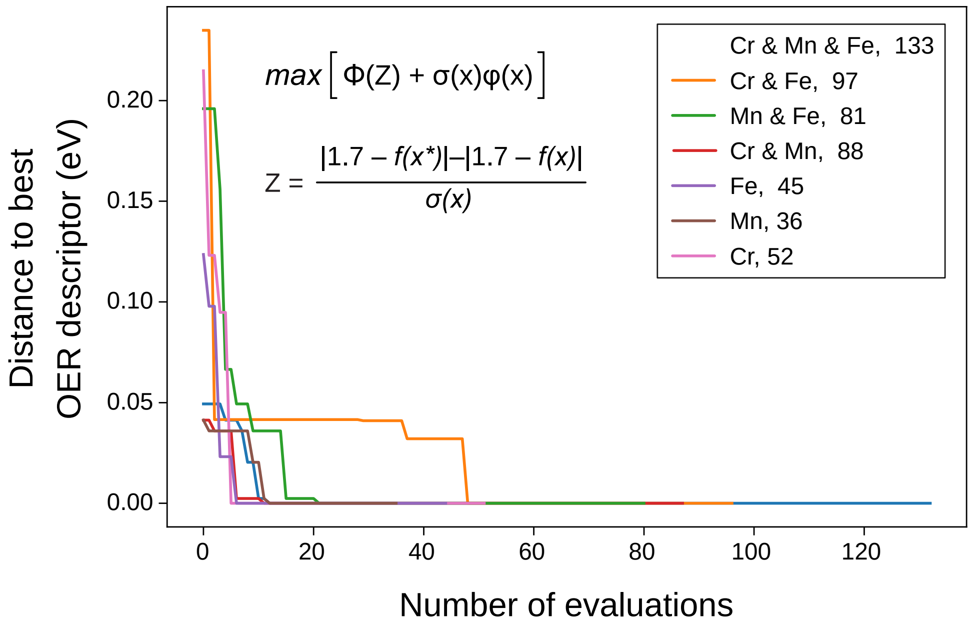

Figure S6. Bayesian optimization over sets of catalysts as in Figure 2b, whereas here the acquisition function that was maximized was the expected improvement as opposed to the probability of improvement acquisition function.

Metal-dependent scaling relations

The m_M values are derived from oxidation state-independent scaling relations between HO* and O* intermediates, where we have focused on Mn and Fe to demonstrate the correlation between $\Delta G_{O(V)^*} - \Delta G_{O(IV)^*}$ and $m_{m.}(\Delta G_{HO(IV)^*} - \Delta G_{HO(III)^*})$. Those scaling relations are shown below.

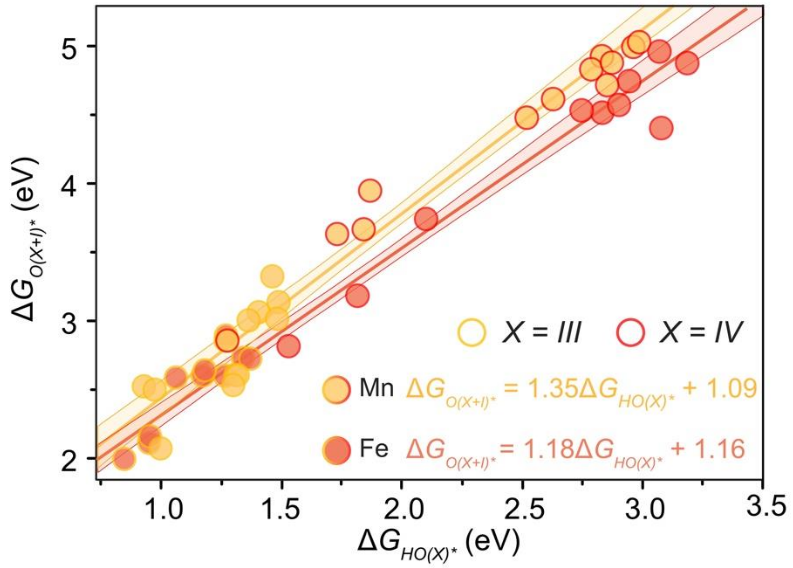

Figure S7. Oxidation state-independent scaling relations for Mn and Fe as in Ref. 3 from the main text, allowing for the use of active learning for low theoretical overpotential.

Model sensitivity to input geometries

To test the generalizability of our approach to distinct starting geometries, we have taken the optimized cartesian coordinates to build another set of feature vectors with which to train and test our machine learning model. The vector which is most different is a Ru-based complex based on the Oct geometry, which is composed of the flexible 4a ligand (shown in Figure 1), and thereby there is a difference in the pre- and post-optimized structure to influence the RACs. However, ultimately, the following data obtained upon repeating the analyses applied to the molSimplify-generated structures show that the overall approach still works in a very similar manner, showing similar errors.

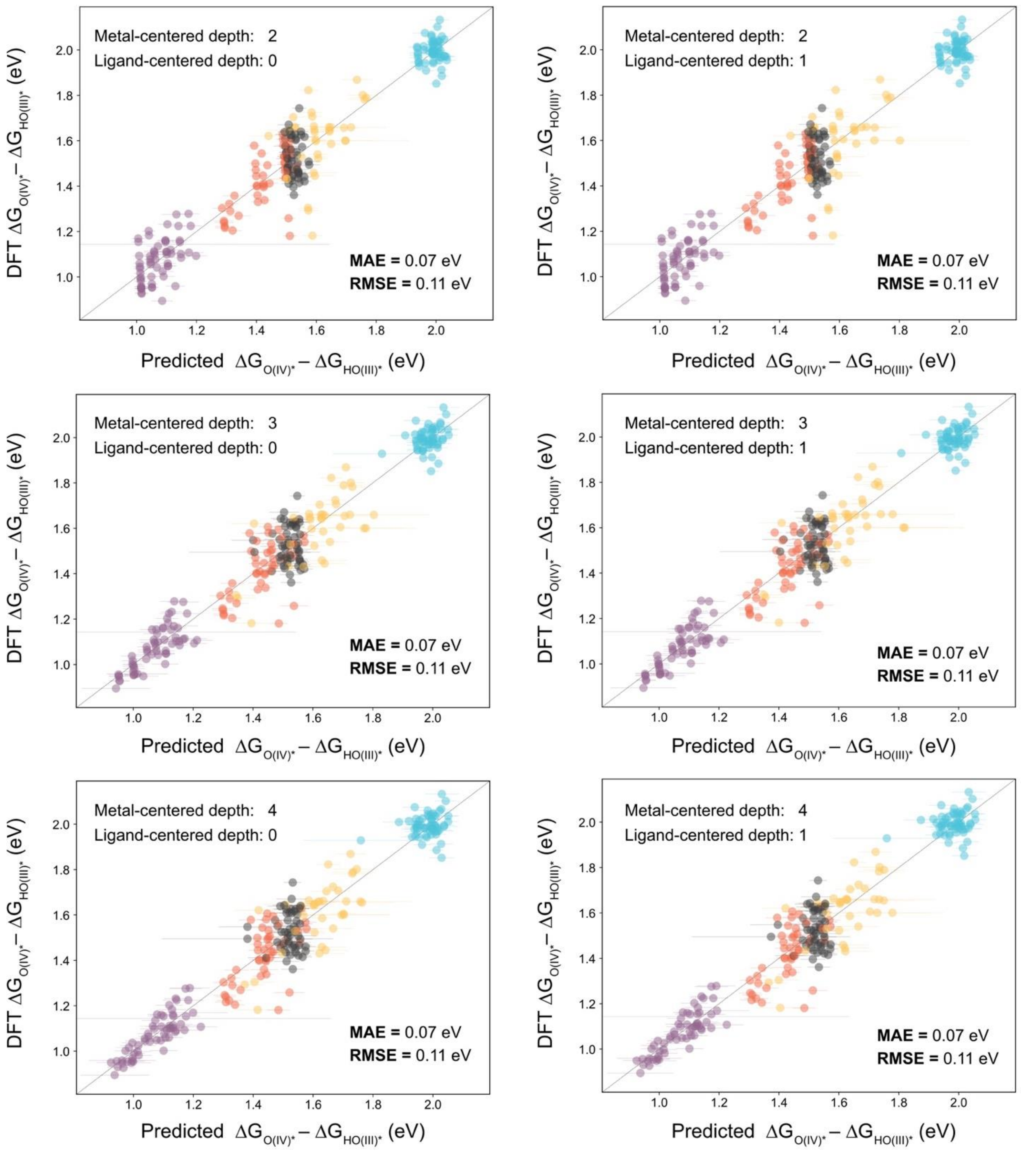

Figure S8. Results for the grid search on molecules with feature vectors created using the TPSSh-optimized geometries.

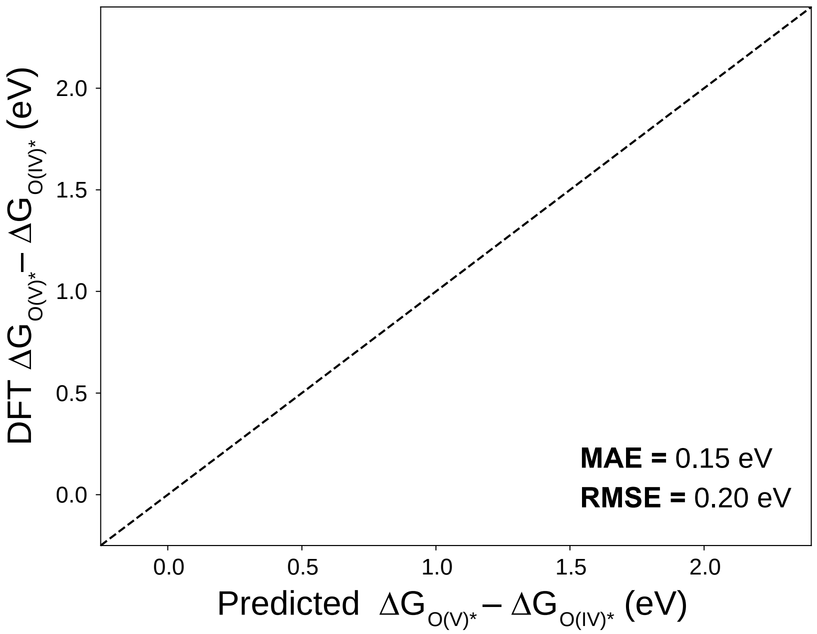

Figure S9. Parity plot for the extra oxidation descriptor as in Figure 4 in the main text while taking the TPSSh-optimized geometries to create the feature vectors.

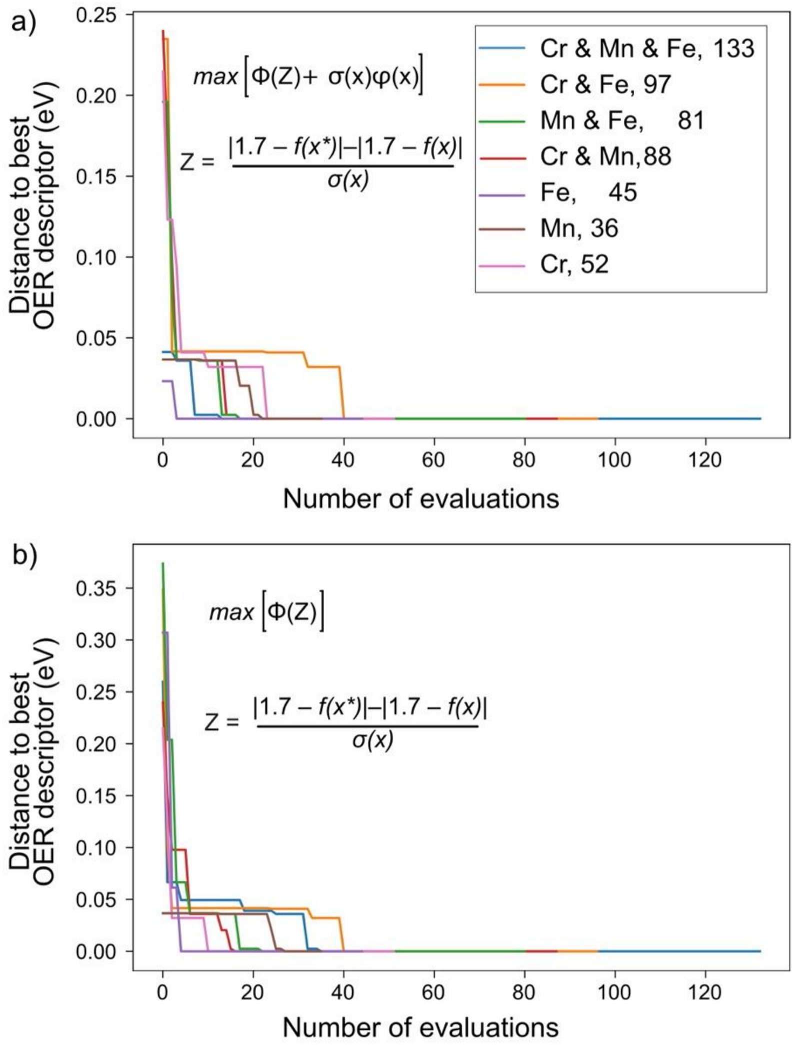

Figure S10. Bayesian optimization using the GPR model trained on TPSSh-optimized geometries with the a) expected improvement and b) probability of improvement acquisition functions.

Cartesian coordinates and energies

All cartesian coordinates, including visualized optimized geometries and energies for each modelled intermediate can be found in the following ioChem-BD dataset: https://iochem-bd.bsc.es/browse/handle/100/198436
